# 

**Published:** 1908-08-15

**Authors:** 


Telegraphic Address: Ospedale, London.

THE MODERN MEDICAL JOURNAL.

Telephone Number: 2734 Gerrard.

NEW SERIES. No. 75, Vol. III. No. 1147. Vol. XLIV.

SATURDAY, AUGUST 15TH, 1908.

PRICE THREEPENCE

